# The new gestational diabetes: Treatment, evidence and consent

**DOI:** 10.1111/ajo.13116

**Published:** 2020-06-07

**Authors:** Christopher K. Hegerty

**Affiliations:** ^1^ Queensland Health Warwick Hospital Warwick Queensland Australia

**Keywords:** informed consent, gestational diabetes, evidence‐based practice, overtreatment, overdiagnosis

## Introduction

Gestational diabetes mellitus (GDM) diagnosis and treatment is now a major part of antenatal care worldwide.

Gestational diabetes is a poorly defined entity with different countries having different diagnostic approaches. In Australia the rate of diagnosis and the use of drugs for treatment is increasing, in part due to the adoption of the liberal International Association of Diabetes and Pregnancy Study Groups diagnostic criteria. Queensland data are presented (Table 1).

**Table 1:** Total births and births to mothers with gestational diabetes mellitus (GDM)

Data items	2012	2013	2014	2015	2016	2017p
Total births	43 694	43 327	44 487	43 743	45 038	43 610
Births with GDM (%)	3206 (7.3)	3551 (8.2)	4090 (9.2)	5165 (11.8)	5949 (13.2)	6290 (14.4)
Treatment: diet (% of GDM births)	1926 (60)	2059 (58)	2338 (57)	2914 (56)	3285 (55)	3310 (53)
Treatment: drug (% of GDM births)	1279 (40)	1489 (42)	1751 (43)	2246 (44)	2659 (45)	2972 (47)

Singleton Births, Public Hospitals, Queensland, 2012–2017. P, preliminary.

Despite the increasing numbers of women diagnosed, there is little to suggest outcomes are improved.^1^, ^2^

In response to these developments this article argues that:

1. evidence‐based benefits from treatment are much less than is generally believed
2. while few babies can benefit, all babies treated, particularly pharmacologically, are exposed to potential harm
3. all treated babies have their growth and lean mass reduced, which may be detrimental, particularly as the majority are already of normal or small size
4. the pharmacological intensification of treatment is not based on sound evidence, is probably unnecessary, potentially harmful, and should be ceased until there is better evidence for benefit and safety, and
5. if parents are not correctly informed about these matters they cannot properly consent to GDM‐related interventions in their otherwise normal pregnancies, and this becomes important if interventions lead to problems.

## What Parents are Told

In Australia young mothers are told that GDM is an ‘epidemic posing an immediate threat’ (Diabetes Australia) but that treatment will reduce miscarriages, stillbirth, premature birth, forceps delivery, ‘larger and fatter’ babies, ‘difficult and dangerous’ births, neonatal breathing problems, feeding problems, temperature problems, and maternal and child diabetes later in life (Queensland and NSW Government provided patient information).

In the United Kingdom (UK) women are told that treatment will reduce ‘serious health problems’, induction of labour, stillbirth, neonatal hypoglycaemia and injuries to the mother and baby (Royal College of Obstetricians and Gynaecologists, National Institute for Health and Care Excellence).

The United States of America (USA) impresses upon mothers that they need to ‘start treatment quickly’ or GDM will ‘hurt you and your baby’ (American Diabetes Association), but that treatment will reduce or eliminate babies ‘wedged in the birth canal’, seizures, threats to the life of the mother (Mayo Clinic), severe tears between the vagina and anus, stress on the heart and kidneys, haemorrhage, shoulder damage, stillbirth (American College of Obstetricians), nerve injuries, broken bones and brain damage (Centers for Disease Control and Prevention).

This information, while startling, is not evidence‐based.

## Are GDM Babies ‘Big and Fat’?

Gestational diabetes babies are about 100–200 g heavier on average,^3^ and in only a small number of macrosomic babies is GDM the cause.^4^

**Figure 1:**
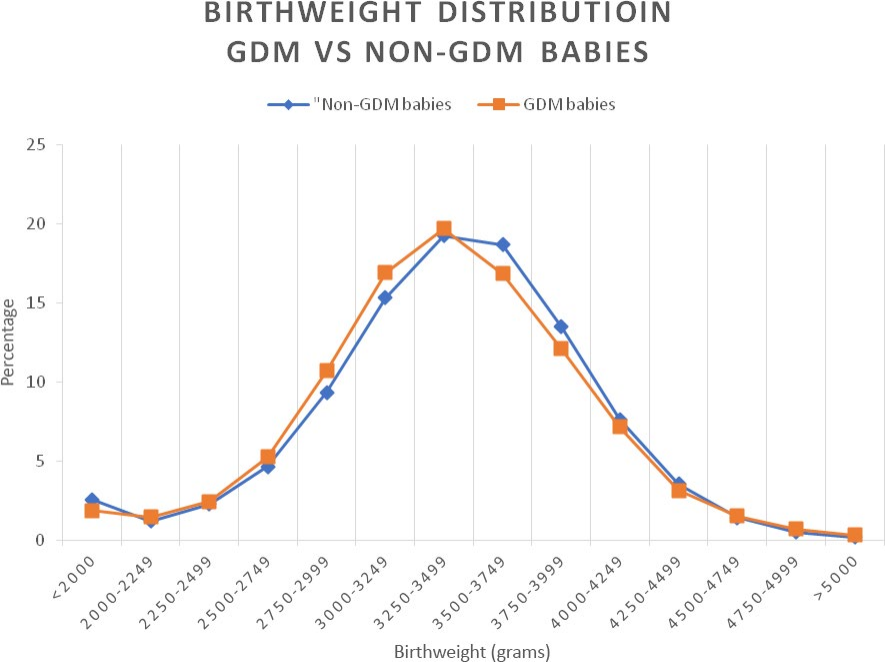
Singleton births, public hospitals, Queensland, 2012–2013, *n* = 87 021. Source: Queensland Perinatal Data Collection, Statistical Services Branch, Dept. of Health, Queensland. GDM, gestational diabetes mellitus

Queensland data illustrate how little different these babies are (Fig. 1).

## The Effect of Treatment

Reviews (which include the two major treatment trials) suggest that glucose control does not benefit babies.

They show three effects:^4^, ^5^, ^6^

1. an average birthweight reduction of 110 g
2. for every 48 women treated one fewer baby will have manoeuvres more than mild traction for delivery (but no difference in harm to babies), and
3. reduced hypertension.

A 2018 Cochrane review concluded that the only likely treatment outcomes are fewer large babies, but at the cost of increased labour inductions, and with no proven benefit from adding pharmacology to lifestyle measures.^7^

Therefore, unless simply reducing a baby’s growth by 110 g is considered beneficial, there seems to be no advantage for babies. Importantly, the birthweight reduction which occurs is mostly lean mass in normal‐sized babies.^8^

Neither insulin, oral hypoglycaemics, nor intense glucose control is shown to improve outcomes compared with diet and exercise alone,^9^, ^10^ and a reduction in hypertensive disorders can reasonably be explained by weight control rather than glucose control.

Glucose levels short of frank diabetes do not increase perinatal mortality,^11^ nor does treatment reduce it,^4^, ^6^, ^7^ and pregnancy hyperglycaemia has not been shown to cause, or treatment reduce, future diabetes or obesity in mother or child.^4^, ^7^, ^12^, ^13^ Treatment does not reduce injury to mothers or babies, forceps delivery, respiratory problems, or neonatal hypoglycaemia, and probably does not reduce caesarean section rates.^4^, ^5^, ^6^, ^7^ A reduction in caesarean section rates occurred in one trial, but this has not been convincingly reproduced in practice. It is possible that restricting the nutrition, growth and size of any babies, GDM or not, reduces the chance of a caesarean section.

Surprisingly, treatment may not benefit girls at all,^8^ and may increase the size of babies of normal‐weight mothers.^14^

Nevertheless, if the process is pleasant, inexpensive and free from harm, perhaps a lack of benefit doesn’t matter.

## Can Treatment be a Disadvantage?

### For mothers

A diagnosis of GDM medicalises a normal pregnancy, moves care away from general practitioners or midwives into the hospital system, triggers interventions such as labour induction, and requires many rural women to have their babies in regional hospitals far from home. Mothers may experience anxiety and a loss of normality and personal control, more so if put on medication. Induction of labour may lead to a less positive birth experience, and self‐perceived health status can be adversely affected. There are extra antenatal visits, work, travel, childcare and financial implications, unpleasant testing, frequent blood sugar measurements, insulin injections, and medication side effects.

### For babies

Reviews of millions of births show no significant increase in perinatal problems until birthweights exceed 4500 g (the top 2% of babies),^15^ but even if there is a benefit in reducing the growth of extremely large babies, the great majority of treated babies are not extremely large.

However, medication is used to reduce the growth of babies of all sizes (Table 2).

**Table 2:** Proportion of babies of gestational diabetes mellitus mothers at different birthweights in whom pharmacology is used

Birthweight (g)	Proportion of babies at this weight in whom pharmacology is used (%)
<2500	36
2500–2999	41
3000–3499	42
3500–3999	41
>4000	43

Singleton Births of mothers with Gestational Diabetes, Public Hospitals, Queensland, 2012–2013, *n* = 6757.

Could this practice be harmful?

## Possible Disadvantages of Nutritional Restriction

There are advantages associated with increasing size, and disadvantages with decreasing size, across the full birthweight range.

Larger birthweight may reflect an optimum intrauterine nutritional environment, and this is consistent with reported improved childhood intelligence, reduced cerebral palsy, and reduced metabolic complications later in life.^16^ Decreasing birthweight across the normal range is associated with increased adult type 2 diabetes, hypertension, cardiovascular disease, stroke, and decreased bone mineral content in adolescence.

Higher birthweight may protect against perinatal death, with USA, European and Australian records showing the lowest perinatal mortality in babies around 4000 g, and with survival progressively worsening as babies’ weights drop below the 75th percentile.^16^

## Reducing Lean Mass

Birthweight correlates more with fat‐free mass than with fat mass in most babies, and the reduction from GDM treatment is composed more of lean mass than fat.^8^ Larger babies are taller as children, have less fat but increased lean mass and height in adolescence and adulthood, and more bone density and muscle mass in old age, so why restrict their growth?

## Drug Effects

Both insulin injections and oral metformin are used to treat GDM, despite a lack of convincing evidence that drugs are necessary or beneficial in addition to lifestyle measures,^7^, ^9^, ^10^ but can drugs sometimes be harmful?

Women on insulin may have frequent nocturnal hypoglycaemic episodes,^17^ so their babies also will suffer recurrent hypoglycaemia, and strict glucose control has been associated with intrauterine growth restriction.^18^ Very low HbA_1c_ levels in pregnant type 1 diabetics have been associated with adverse outcomes, and already small babies of GDM mothers have died in a number of studies while having their growth pharmacologically restricted. While no causal implication is possible, this is concerning. Insulin also makes it harder for mothers to control weight gain, may be associated with delayed milk production, and may increase blood pressure.^7^, ^19^

Metformin, a Category C drug with no long‐term safety data, readily crosses the placenta, resulting in levels in babies similar to those in adult diabetics.^20^ Effectively the baby is being given therapeutic metformin. It causes gastrointestinal side effects in mothers (and possibly babies), and women may lose less weight post‐partum. Children exposed to metformin *in utero* may have altered growth patterns, including having larger heads or being shorter at birth,^21^ and may have long‐term metabolic changes including higher childhood obesity rates.^22^

## Conclusion

A diagnosis of pregnancy hyperglycaemia affects about 21 million women worldwide each year, and unless these women are correctly informed by their carers they cannot effectively consent to testing or treatment. Importantly, for most babies, the evidence for treatment benefit is thin.

Parents should be fully and correctly informed about their baby’s chance of treatment benefit, neither overstating advantages nor understating disadvantages, and testing could be presented as an option rather than as a requirement to properly informed parents, some of whom may decline.

Nutrition and growth have advantages in the short and long term, and benefits may decrease with decreasing birthweight, so growth should be restricted only for clear reasons, particularly in normal or small babies.

Intensification of treatment with drugs has not been shown to be necessary in addition to lifestyle measures, may have unwanted effects, and cannot be shown to be safe. It has traditionally been the practice with drug use in pregnancy to be cautious, and to only use drugs if the benefits clearly outweigh the potential, possibly unknown, harms.

I suggest that in gestational diabetes treatment this precautionary principle would at present preclude the use of pharmacology until such a time as well‐run clinical trials provide better evidence for safety, necessity and effectiveness.
